# Reframing Heart Failure as a Multiorgan Network Disorder: Translational and Regenerative Perspectives in Veterinary Cardiology

**DOI:** 10.3390/vetsci13050435

**Published:** 2026-04-29

**Authors:** Mitsuhiro Isaka, Hiromu Udagawa, Yuji Hamamoto, Eunryel Nam

**Affiliations:** Laboratory of Companion Animal Surgery, School of Veterinary Medicine, Rakuno Gakuen University, Ebetsu 069-8501, Hokkaido, Japany-hamamoto@rakuno.ac.jp (Y.H.); er-nam@rakuno.ac.jp (E.N.)

**Keywords:** heart failure, multiorgan crosstalk, myxomatous mitral valve disease, dilated cardiomyopathy, translational veterinary cardiology

## Abstract

Heart failure (HF) has traditionally been regarded as a primary myocardial disorder in veterinary medicine. However, accumulating evidence suggests that HF represents a systemic syndrome characterized by dynamic multiorgan interactions. In human cardiovascular research, cardiorenal and cardiointestinal paradigms have reshaped disease conceptualization, yet comparable integrative frameworks remain underdeveloped in veterinary cardiology. Naturally occurring canine HF—particularly myxomatous mitral valve disease and dilated cardiomyopathy—offers a clinically relevant translational platform in which systemic remodeling unfolds within an intact physiological lifespan. This review proposes a systems-based perspective that integrates spontaneous canine HF with controlled in vivo experimental models. We outline four main pathways of interaction: (1) the heart–gut axis, wherein reduced perfusion can influence inflammation and disruption of the intestinal barrier; (2) the heart–bone axis, wherein endocrine factors like osteoprotegerin and osteocrin can impact remodeling of the cardiovascular system; (3) the heart–vascular endothelium axis, wherein inflammatory signaling and dysfunction of the vascular endothelium are hallmarks; and (4) the neurocardiac axis, which reflects an imbalance in the autonomic nervous system. Emerging regenerative and organelle-based strategies—including mesenchymal stem cell therapy and mitochondrial transplantation—are discussed within this multiorgan framework. Rather than focusing solely on cardiac contractility, these approaches may function as systemic inflammatory modulators, and endothelial, metabolic, and autonomic pathways. Canine HF can be better understood as a multiorgan network condition; reframing it in this way can help researchers in the field of translational cardiology create more comprehensive diagnostic and treatment plans.

## Simple Summary

Heart failure (HF) in dogs has traditionally been viewed as a disease limited to the heart. However, growing evidence suggests that HF is a systemic condition involving complex interactions among multiple organs. In this review, we propose that canine HF—particularly myxomatous mitral valve disease and dilated cardiomyopathy—should be understood as a multiorgan network disorder rather than an isolated cardiac problem. We describe four major interaction pathways: (1) the heart–gut axis, where reduced circulation may disrupt intestinal barrier function and promote inflammation; (2) the heart–bone axis, involving endocrine factors such as osteoprotegerin and osteocrin that may influence cardiovascular remodeling; (3) the heart–vascular endothelium axis, characterized by vascular dysfunction and inflammatory signaling; and (4) the neurocardiac axis, reflecting autonomic nervous system imbalance. Naturally occurring heart disease in dogs provides a valuable translational model because it develops spontaneously within an intact physiological lifespan. When combined with controlled experimental models, this approach allows investigation of how cardiac dysfunction influences other organs and vice versa. We also discuss emerging regenerative strategies, including mesenchymal stem cell therapy and mitochondrial transplantation. Rather than acting solely on cardiac contractility, these therapies may modulate systemic inflammation, endothelial function, metabolism, and autonomic balance. Reframing canine HF as a multiorgan network disorder may improve diagnostic strategies, refine therapeutic approaches, and strengthen the translational bridge between veterinary and human cardiovascular medicine.

## 1. Introduction

Heart failure (HF) has traditionally been defined as a clinical syndrome resulting from impaired cardiac function. However, accumulating evidence from human medicine has established that HF is not merely a myocardial disorder but a complex systemic condition characterized by dynamic interactions among multiple organs, including the kidneys, gastrointestinal tract, musculoskeletal system, and vascular endothelium [1,2,3].

Concepts such as cardiorenal and cardiointestinal syndromes have reshaped contemporary understanding of HF pathophysiology, emphasizing bidirectional signaling pathways, inflammatory activation, neurohormonal dysregulation, and metabolic remodeling. In veterinary medicine, naturally occurring HF in dogs—most commonly secondary to myxomatous mitral valve disease (MMVD) and dilated cardiomyopathy (DCM)—shares many clinical and pathophysiological features with human HF, including neurohormonal activation, endothelial dysfunction, and systemic congestion. The aging canine population and the high prevalence of chronic valvular disease provide a unique opportunity to investigate HF as a spontaneous, heterogeneous, and clinically relevant condition. Nevertheless, the systemic nature of canine HF remains insufficiently integrated into a unified conceptual framework [3,4,5,6,7,8,9,10,11,12,13,14,15,16,17,18,19,20,21,22,23,24,25,26,27,28,29,30,31].

Recent studies in dogs have suggested that HF is associated with alterations beyond the myocardium, including intestinal barrier dysfunction, changes in gut microbiota composition, and circulating biomarkers reflecting extra-cardiac organ involvement. In parallel, emerging data indicate potential interactions between cardiac dysfunction and skeletal signaling pathways, including regulatory peptides implicated in bone and metabolic homeostasis. Furthermore, endothelial activation and inflammatory signaling appear to contribute significantly to HF progression, particularly in settings involving circulatory stress or surgical interventions [9,10,11,12,13,14,15,16,17,18,24,27,28,29,30,32,33,34,35,36].

Experimental in vivo models remain essential for elucidating mechanistic pathways underlying these organ interactions. Anthracycline-induced dilated cardiomyopathy models, surgical ventricular reconstruction approaches, and cardiopulmonary bypass systems have provided controlled platforms to investigate myocardial injury, endothelial dysfunction, and systemic inflammatory responses. When integrated with observations from naturally occurring canine HF, these models offer a translational bridge between veterinary cardiology and comparative medicine [17,18,28,29,30,37,38,39,40,41].

Accordingly, we propose that canine HF should be conceptualized not as an isolated myocardial disorder but as a dynamic multiorgan network disease characterized by bidirectional signaling among cardiac, intestinal, skeletal, endothelial, and neural systems [9,10,11,12,13,14,15,16,17,18,24,25,26,27,28,29,30,37,38,39,40,41,42].

In our previous experimental and clinical investigations, we have observed coordinated alterations in biomarkers reflecting intestinal injury, bone-derived signaling, and endothelial activation in both spontaneous canine heart disease and controlled cardiomyopathy models. These findings support the hypothesis that HF represents a system-level disorder rather than an organ-confined pathology. Integrating naturally occurring canine HF with mechanistic in vivo models provides a unique translational framework to dissect causal pathways and identify systemic therapeutic targets. This network-based perspective may redefine disease staging and open up new avenues for regenerative and multiorgan-directed interventions in veterinary cardiology and comparative cardiovascular medicine [9,10,11,12,13,14,15,16,17,18,24,25,26,27,28,29,30,32,33,34,35,36,37,38,39,40,41,43,44,45,46,47,48].

## 2. Canine HF as a Naturally Occurring Translational Model

Naturally occurring HF in dogs represents a clinically relevant and biologically complex model that parallels several aspects of human HF. Because of the age-related effects, neurohormonal activation, and systemic remodeling that accompany these disorders, canine HF is an excellent model for examining the development of heart failure throughout time [4,5,6,7,8,19,20,21,22,23,31,49]. The most common kind of canine-acquired heart disease, known as mitral valve degeneration (MVD), causes left ventricular and atrial remodeling as well as congestive heart failure (HF) in the long run. A combination of sympathetic stimulation, inflammatory signaling, and activation of the renin–angiotensin–aldosterone system (RAAS) leads to structural and functional degradation, much like in human valvular heart disease. Among the companion animals, dogs most commonly develop HF secondary to MMVD and DCM, conditions that collectively account for the majority of chronic cardiac morbidity in small and large breeds, respectively. The progressive nature of these diseases, combined with the influence of aging, neurohormonal activation, and systemic remodeling, makes canine HF particularly valuable for studying the longitudinal evolution of cardiac dysfunction [4,5,6,7,8,19,20,21,22,23,31,49]. MMVD, the most prevalent acquired cardiac disease in dogs, is characterized by progressive valvular degeneration, cardiac remodeling, and eventual congestive HF. Similarly to human valvular heart disease, activation of the renin–angiotensin–aldosterone system (RAAS), sympathetic stimulation, and inflammatory signaling contribute to disease progression [4,5,6,7,8,17,18,19,28,29,30,37,38,39,40,41,50,51,52,53,54,55,56,57,58]. Canine DCM similarly shares key features with human nonischemic cardiomyopathy, including ventricular dilation, systolic dysfunction, and arrhythmogenesis [20,21,22,23,31,49,59,60,61,62]. Beyond myocardial dysfunction, dogs with HF exhibit systemic manifestations such as gastrointestinal disturbances, endothelial activation, skeletal signaling alterations, and changes in circulating biomarkers [4,5,6,7,8,9,10,11,12,13,14,15,16,17,18,19,24,25,26,27,28,29,30,32,33,34,35,36,37,38,39,40,41]. These findings support the concept that canine HF reflects a multiorgan condition rather than an isolated cardiac disorder. However, while associative clinical data are increasing, mechanistic pathways underlying these multiorgan interactions remain incompletely defined. In particular, it is still unclear whether extracardiac alterations—such as intestinal barrier dysfunction, endothelial activation, or bone-derived signaling changes—represent primary drivers of disease progression, adaptive responses, or secondary consequences of hemodynamic impairment [9,10,11,12,13,14,15,16,17,18,20,21,22,23,24,25,26,27,28,29,30,36,39,40,41].

The spontaneous and heterogeneous nature of canine HF enhances its translational relevance, as clinical populations reflect real-world variability in genetics, comorbidities, and environmental influences. At the same time, this heterogeneity can limit mechanistic interpretation and causal inference. Therefore, integration with controlled experimental in vivo models remains essential to elucidate underlying signaling pathways and temporal relationships across organ systems [31,37,38,39,40,41,46,63].

Taken together, naturally occurring canine HF provides a valuable translational platform for investigating systemic disease processes. Nevertheless, further studies combining clinical phenotyping with mechanistic experimentation are required to clarify the causal pathways linking cardiac dysfunction to multiorgan remodeling and to identify potential system-level therapeutic targets.

## 3. Experimental In Vivo Models Enabling Mechanistic Insights into Multiorgan Crosstalk

While naturally canine HF provides indispensable clinical relevance, controlled experimental in vivo models remain essential for dissecting mechanistic pathways underlying multiorgan interactions.

Clinical heterogeneity, comorbidities, and therapeutic variability in spontaneous cases often limit causal inference. Experimental systems therefore complement clinical observations by enabling temporal control of injury, standardized interventions, and mechanistic interrogation [37,38,39,40,41].

Among established platforms, anthracycline-induced cardiomyopathy models have been widely used to reproduce DCM-like phenotypes characterized by progressive systolic dysfunction, ventricular dilation, and myocardial remodeling. Doxorubicin–or daunorubicin-induced models replicate the key features of oxidative stress, mitochondrial dysfunction, and cardiomyocyte apoptosis observed in nonischemic cardiomyopathy. Importantly, these models permit longitudinal evaluation of systemic alterations, including inflammatory activation and metabolic disturbances, thereby providing a framework for investigating heart–organ signaling beyond primary myocardial injury. Experimental DCM models also enable exploration of circulating biomarkers reflecting extracardiac involvement. Studies have demonstrated alterations in markers associated with intestinal injury, endothelial activation, and bone-related signaling in cardiomyopathy contexts. By establishing controlled timelines of myocardial dysfunction, these systems allow investigation of whether such biomarkers represent secondary consequences of congestion and hypoperfusion or active contributors to disease progression [9,10,11,12,13,14,15,16,17,18,20,21,22,23,24,25,26,27,28,29,30,31,32,33,34,35,36,37,38,39,40,41,49,59,60,61,62].

Surgical and circulatory intervention models further expand mechanistic understanding. Cardiopulmonary bypass (CPB) systems, for example, provide a controlled platform in which perfusion mode (pulsatile vs. non-pulsatile), duration of extracorporeal circulation, temperature management, and ischemia–reperfusion timing can be systematically manipulated [37,38,39,40,41]. These models reproducibly induce systemic inflammatory responses and endothelial activation through ischemia–reperfusion injury and contact-mediated immune stimulation associated with blood–surface interactions [39,40,41,64,65]. Endothelial dysfunction, characterized by altered expression of vasoactive mediators such as ET-1 and components of the RAAS, contributes to vascular instability and multiorgan stress [17,18,28,29,30,56].

Importantly, CPB models allow temporal assessment of circulating and tissue-level responses, including cytokine release, leukocyte activation, endothelial permeability, and microvascular dysfunction, thereby enabling mechanistic evaluation of how acute hemodynamic perturbations propagate to extracardiac organs [39,40,41,64,65]. In addition, these systems facilitate investigation of the heart–vascular endothelium axis under defined conditions and provide insight into how endothelial activation may serve as a central mediator linking cardiac injury to systemic inflammation and organ dysfunction [17,18,28,29,30]. As such, CPB-based intervention models represent a valuable experimental framework for dissecting causal pathways underlying multiorgan crosstalk in HF.

Importantly, in vivo models facilitate evaluation of therapeutic modulation targeting organ crosstalk. Pharmacologic interventions, including neurohormonal blockade and receptor-specific agents, can be administered under controlled conditions to assess effects on myocardial performance, inflammatory signaling, and extracardiac biomarkers. Non–pharmacologic strategies, such as exercise-based or neuromuscular stimulation paradigms, may also influence systemic metabolic and skeletal muscle pathways implicated in HF progression. Experimental platforms thus serve not only as tools for reproducing cardiac dysfunction but also as translational bridges for testing multiorgan-targeted strategies [37,38,39,40,41].

The integration of spontaneous canine HF and complementary in vivo systems represents a bidirectional translational approach. Clinical observations generate hypotheses regarding systemic involvement, while controlled models enable mechanistic validation and therapeutic refinement. This iterative framework strengthens the conceptualization of HF as a systemic disorder and positions veterinary research as a contributor to comparative cardiovascular science [37,38,39,40,41].

## 4. Organ Crosstalk Axes in Canine and Experimental HF

### 4.1. The Heart–Gut Axis

**Figure 1:**
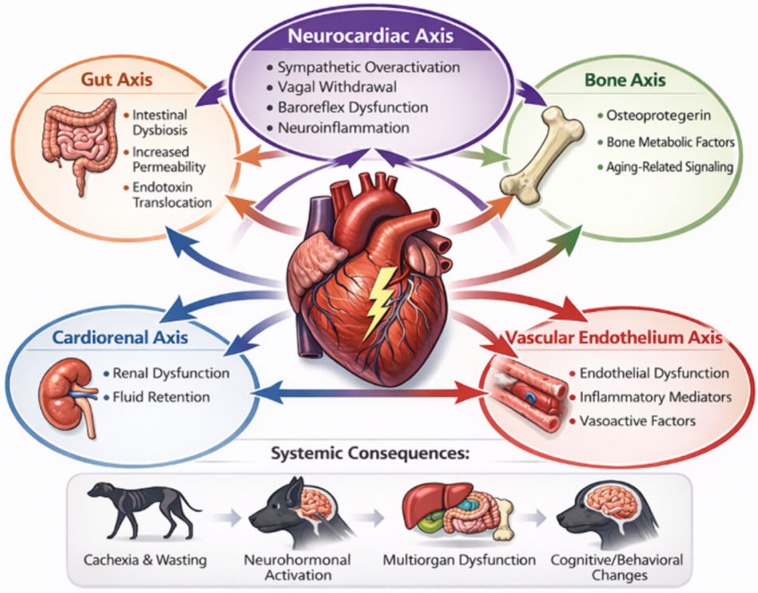
Integrated network model of multiorgan crosstalk in chronic heart failure. Schematic representation of the failing heart as a central node within a dynamic multiorgan interaction network. Bidirectional signaling occurs between the myocardium and peripheral organ systems, including the gastrointestinal tract (heart–gut axis), skeletal system (heart–bone axis), vascular endothelium (heart–vascular axis), and central/autonomic nervous system (neurocardiac axis). Reduced cardiac output and venous congestion contribute to intestinal barrier dysfunction and microbial translocation, amplifying systemic inflammation. Bone-derived mediators such as osteoprotegerin and osteocrin participate in endocrine and vascular signaling. Endothelial activation and vasoactive mediators modulate organ perfusion and inflammatory amplification. Autonomic imbalance, characterized by sympathetic overactivation and vagal withdrawal, links cardiac dysfunction to arrhythmogenesis and neuroimmune modulation.

The concept of a heart–gut axis has gained increasing attention in human HF research, where intestinal congestion, hypoperfusion, and barrier dysfunction contribute to systemic inflammation and disease progression. Reduced cardiac output and elevated venous pressure impair splanchnic circulation, leading to mucosal ischemia, increased intestinal permeability, and translocation of endotoxins and microbial metabolites. These processes activate inflammatory pathways that may exacerbate myocardial remodeling (Figure 1) [9,10,11,12,13,14,15,16,19,24,25,26,27,50,51,52,53,54,55,56,57,58].

These axes are interconnected through shared pathways including neurohormonal activation, oxidative stress, and inflammatory signaling, collectively driving disease progression and systemic remodeling. This schematic illustration was created by the authors using AI-assisted graphic tools and modified for this study.

In canine HF, emerging evidence suggests similar pathophysiological mechanisms. Dogs with MMVD have been reported to exhibit gastrointestinal complications and alterations in intestinal microbiota composition, indicating that chronic cardiac dysfunction may influence gut homeostasis. Although causal relationships remain to be fully elucidated, intestinal barrier disruption may represent both a consequence and a contributor to systemic inflammation in canine HF [4,5,6,7,8,9,10,11,12,13,14,15,16,19,24,25,26,27].

Circulating biomarkers reflecting enterocyte injury, such as intestinal fatty acid–binding protein (I–FABP), have been investigated in cardiomyopathy contexts. Elevations in such markers may signal subclinical mucosal damage secondary to congestion or hypoxia. Experimental DCM models further provide opportunities to determine whether gut-derived inflammatory mediators precede or follow overt cardiac decompensation [9,10,11,12,13,14,15,16,20,21,22,23,24,25,26,27,31,49].

The heart–gut axis also presents potential therapeutic implications. Modulation of gastrointestinal inflammation, microbial composition, or histamine–mediated signaling pathways may influence systemic inflammatory tone and cardiac remodeling. Controlled experimental systems are particularly valuable for evaluating whether targeting intestinal dysfunction can attenuate myocardial progression [9,10,11,12,13,14,15,16,24,25,26,27,37,38,39,40,41].

In veterinary medicine, clinical evidence supporting the heart–gut axis has begun to emerge. In a cohort of Chihuahuas with MMVD, intestinal complications and alterations in fecal microbiota composition were observed, suggesting that chronic cardiac remodeling may influence gastrointestinal homeostasis. Notably, changes in bacterial diversity and relative abundance were associated with disease severity, raising the possibility that progressive cardiac dysfunction may induce microbial shifts through venous congestion, reduced perfusion, or neurohormonal activation. These findings provide a clinical foundation for conceptualizing canine HF as a disorder involving intestinal barrier and microbial dysregulation rather than isolated myocardial failure [4,5,6,7,8,9,10,11,12,13,14,15,16,19,24,25,26,27].

Complementing clinical observations, experimental anthracycline-induced rabbit models of DCM have demonstrated significant increases in circulating I–FABP, a sensitive marker of enterocyte injury, in parallel with declining systolic function. Importantly, these alterations occurred in the absence of overt gastrointestinal disease, suggesting subclinical mucosal compromise secondary to cardiac dysfunction. The temporal association between ventricular remodeling and biomarker elevation supports the hypothesis that intestinal barrier dysfunction may represent an early systemic consequence of cardiomyopathy rather than merely a terminal event [9,10,11,12,13,14,15,16,20,21,22,23,24,25,26,27,31,49].

### 4.2. The Heart–Bone Axis

Beyond traditional neurohormonal mechanisms, HF has increasingly been recognized as a disorder involving endocrine and skeletal signaling pathways. Bone-derived factors, including osteoprotegerin (OPG) and other regulatory peptides, have been implicated in vascular calcification, inflammation, and cardiovascular risk in humans. These mediators function not only in skeletal metabolism but also in vascular and myocardial remodeling [17,32,33,34,35,36].

In veterinary medicine, circulating bone-related markers have been evaluated in aging dogs and in cardiac disease contexts. Although data remain limited, alterations in such mediators suggest potential bidirectional communication between cardiac dysfunction and skeletal signaling systems. Age-related changes, particularly in small-breed dogs predisposed to MMVD, may further amplify endocrine interactions influencing disease progression [4,5,6,7,8,17,19,32,33,34,35,36,37,38,39,40,41].

The heart–bone axis may involve inflammatory cytokines, oxidative stress pathways, and shared regulatory networks affecting both myocardial and skeletal tissues. Experimental cardiomyopathy models allow investigation of whether bone-associated peptides change in response to controlled myocardial injury and whether such alterations contribute to systemic remodeling. Recognizing these interactions expands the conceptualization of HF beyond hemodynamics and neurohormonal activation [17,32,33,34,35,36,59,60,61,62].

Interestingly, in the same experimental context, circulating OPG concentrations were concurrently altered, raising the possibility of coordinated heart–gut–bone signaling pathways. Given that OPG has been implicated in inflammatory and vascular remodeling processes, simultaneous modulation of I–FABP and OPG suggests a systemic inflammatory network connecting intestinal integrity, endocrine signaling, and myocardial remodeling. This integrative perspective extends the traditional gut–heart concept toward a broader multiorgan interaction model in canine cardiomyopathy [9,10,11,12,13,14,15,16,17,24,25,26,27,32,33,34,35,36].

In aging dogs without overt HF, coordinated elevations in OPG, angiotensin II, and endothelin–1 have been documented, suggesting an interaction between bone-derived mediators and vasoactive neurohormonal pathways. These findings indicate that skeletal and vascular signaling systems may be primed even prior to clinically apparent cardiac decompensation. Given the high prevalence of MMVD in elderly small-breed dogs, age–related endocrine shifts may contribute to susceptibility to myocardial remodeling and progressive HF [4,5,6,7,8,17,18,19,28,29,30,32,33,34,35,36,37,38,39,40,41].

Furthermore, perioperative alterations in circulating osteocrin (OSTN) concentrations have been observed in dogs undergoing orthopedic surgical procedures. Although primarily investigated in the musculoskeletal context, OSTN has been implicated in natriuretic peptide signaling and cardiovascular homeostasis. These observations raise the possibility that skeletal-derived peptides may dynamically respond to systemic stress and influence cardiac signaling pathways. In the context of HF, such endocrine mediators may modulate myocardial remodeling, vascular tone, or inflammatory activation [17,32,33,34,35,36].

The heart–bone axis may therefore represent a bidirectional signaling network involving inflammatory cytokines, oxidative stress pathways, and shared regulatory mediators affecting both skeletal and myocardial tissues. Experimental cardiomyopathy models provide a controlled platform to determine whether the modulation of bone-assisted peptides influence ventricular remodeling trajectories. Integrating endocrine biomarkers into HF phenotyping may refine risk stratification and identify novel therapeutic targets in veterinary cardiology [17,32,33,34,35,36,59,60,61,62].

### 4.3. The Heart–Vascular Endothelin Axis

Endothelial dysfunction is a central component of HF pathophysiology in humans and is increasingly recognized in veterinary cardiology. Impaired nitric oxide bioavailability, activation of ET–1, and upregulation of the RAAS contribute to vasoconstriction, inflammation, and microvascular instability [17,18,28,29,30]. In canine HF, neurohormonal activation and systemic congestion may promote endothelial stress and inflammatory signaling. Circulatory intervention models, such as CPB systems, demonstrate that ischemia–reperfusion injury and systemic inflammatory responses can directly induce endothelial activation and vascular permeability changes. These findings underscore the importance of vascular integrity in modulating multiorgan consequences of cardiac dysfunction [17,18,28,29,30,37,38,39,40,41].

Endothelial injury not only reflects cardiac severity but may actively amplify organ crosstalk by altering tissue perfusion and inflammatory mediator release. Vasoactive peptides such an ET–1 and angiotensin II exert systemic effects influencing renal, intestinal, and skeletal tissues. Thus, the heart–endothelium axis functions as a central conduit through which cardiac pathology propagates systemic alterations [9,10,11,12,13,14,15,16,17,18,24,25,26,27,28,29,30]. Integrating endothelial biology into the framework of canine HF reinforces the view that vascular regulation represents both a mechanistic driver and a therapeutic target in multiorgan disease progression [17,18,28,29,30]. In addition to reflecting the severity of the heart attack, endothelial injury may actively increase organ crosstalk by changing tissue perfusion and the release of inflammatory mediators. Renal, gastrointestinal, and skeletal tissues are all affected by the systemic effects of vasoactive peptides such ET-1 and angiotensin II. Therefore, cardiac disease propagates systemic changes through the heart–endothelium axis [9,10,11,12,13,14,15,16,17,18,24,25,26,27,28,29,30]. By including endothelial biology into the model of canine HF, we may further support the idea that vascular control is a therapeutic target and a mechanism driving the course of multiorgan diseases [17,18,28,29,30].

Collectively, these findings raise the possibility that skeletal-derived mediators participate in the integrated inflammatory–endocrine network linking aging, vascular tone, and myocardial remodeling. From a translational perspective, incorporation of bone-related biomarkers into HF phenotyping may provide mechanistic insights beyond traditional neurohormonal paradigms [17,32,33,34,35,36].

### 4.4. The Heart–Brain (Neurocardiac) Axis

**Figure 2:**
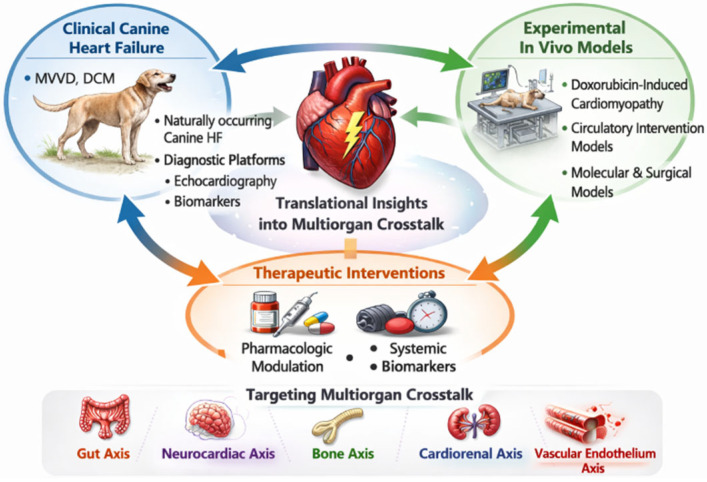
Bidirectional translational framework integrating spontaneous canine heart failure and experimental in vivo models. Naturally occurring canine heart failure, including myxomatous mitral valve disease and dilated cardiomyopathy, provides clinically relevant and heterogeneous disease phenotypes characterized by longitudinal remodeling, neurohormonal activation, and systemic involvement. These spontaneous models allow comprehensive phenotyping through echocardiography, circulating biomarkers, and clinical outcome assessment. Complementary experimental in vivo models, including anthracycline-induced cardiomyopathy and cardiopulmonary bypass systems, enable temporal control of myocardial injury and mechanistic interrogation of endothelial activation, inflammatory signaling, and multiorgan interactions. The integration of clinical observation and experimental validation establishes a bidirectional translational platform. Clinical findings generate mechanistic hypotheses, while controlled models refine causal inference and therapeutic targeting. This iterative framework positions veterinary cardiology as a contributor to comparative cardiovascular science and supports the development of multiorgan-oriented diagnostic and therapeutic strategies. This schematic illustration was created by the authors using AI-assisted graphic tools and modified for this study.

HF is closely associated with dysregulation of the autonomic nervous system and the central autonomic network, forming a bidirectional “neurocardiac axis” that links myocardial dysfunction with neural and immune signaling. In human HF, persistent sympathetic activation and vagal withdrawal contribute to vasoconstriction, arrhythmogenesis, adverse remodeling, and systemic inflammation. This autonomic imbalance is not merely compensatory but becomes maladaptive, accelerating disease progression. In veterinary cardiology, autonomic dysfunction has also been documented in dogs with naturally occurring MMVD (Figure 2).

Heart rate variability (HRV) analyses demonstrate altered sympathovagal modulation compared with healthy controls, and reductions in HRV indices correlate with disease severity and arrhythmogenic profiles. These findings suggest that neurophysiologic remodeling accompanies structural cardiac changes in canine heart disease.

Mechanistically, impaired baroreflex sensitivity and altered chemoreflex function are major contributors to sustained sympathetic excitation in HF. This dysregulation reinforces neurohormonal activation and endothelial dysfunction, linking the neurocardiac axis to vascular inflammation and multiorgan impairment. Importantly, autonomic signaling also influences intestinal permeability and inflammatory responses, thereby intersecting with the heart–gut axis, while neuroimmune pathways may modulate bone remodeling and osteokine signaling, connecting to the heart–bone axis described above. From a translational standpoint, neuromodulatory therapies have been explored in human HF to restore autonomic balance. Baroreflex activation therapy has demonstrated clinical benefit in patients with reduced ejection fraction, and vagus nerve stimulation has been investigated as a strategy to attenuate sympathetic overactivity and inflammation. Although these approaches remain largely experimental in veterinary medicine, canine HF represents a promising comparative model for evaluating whether targeted modulation of autonomic tone can favorably influence arrhythmia burden, endothelial activation, gut-derived inflammation, and systemic progression [4,5,6,7,8,9,10,11,12,13,14,15,16,17,18,19,24,25,26,27,28,29,30,32,33,34,35,36,50,51,52,53,54,55,56,57,58]. Collectively, the neurocardiac axis should be regarded not only as a marker of HF severity but as a potential driver of multiorgan crosstalk and therapeutic target in veterinary translational cardiology.

HF should therefore be considered not only a hemodynamic syndrome but also a disorder of autonomic network remodeling. Persistent sympathetic overactivation and vagal withdrawal constitute a maladaptive regulatory state that propagates vascular dysfunction, arrhythmogenesis, intestinal permeability changes, and systemic inflammation. Within this framework, autonomic imbalance may function as both a driver and amplifier of multiorgan crosstalk, positioning neuromodulation as a potential system-level therapeutic strategy rather than a purely rhythm-forced intervention [9,10,11,12,13,14,15,16,19,24,25,26,27,37,38,39,40,41,50,51,52,53,54,55,56,57,58].

### 4.5. Inter-Axis Crosstalk and Shared Signaling Pathways

While each organ axis provides a useful conceptual framework, these pathways do not operate in isolation. Rather, HF represents an integrated network in which the heart–gut, heart–bone––vascular endothelium, and neurocardiac axes are interconnected through overlapping signaling mechanisms and bidirectional feedback loops [9,10,11,12,13,14,15,16,17,18,24,25,26,27,28,29,30,35,36,39,40,41].

A central feature linking these axes is systemic inflammatory activation. For example, intestinal barrier dysfunction in the heart–gut axis promotes translocation of endotoxins and microbial metabolites, which activate circulating immune pathways [9,10,11,12,13,14,15,16,24,25,26,27]. These inflammatory mediators can directly impair endothelial function, contributing to the heart–vascular axis [17,18,28,29,30], and may also influence bone-derived signaling molecules such as OPG, thereby connecting to the heart–bone axis [17,32,33,34,35,36]. In parallel, chronic inflammation modulates autonomic nervous system activity, reinforcing sympathetic activation and neurocardiac dysregulation [49,50,51,57].

Neurohormonal signaling pathways, particularly RAAS and ET signaling, serve as another major integrative mechanism. Activation of RAAS not only contributes to cardiac remodeling but also affects intestinal perfusion, endothelial tone, and skeletal metabolism [17,18,28,29,30,56]. Angiotensin II and ET–1 have been implicated in vascular dysfunction, inflammatory amplification, and modulation of bone-related mediators [17,18,28,29,30,32,33,34,35,36], suggesting that these pathways act as systemic coordinators of multiorgan responses.

Endothelial dysfunction represents a key interface across axes. The vascular endothelium regulates organ perfusion, leukocyte trafficking, and barrier integrity [17,18,28,29,30]. Endothelial activation may exacerbate intestinal hypoperfusion, promoting gut-derived inflammation [9,10,11,12,13,14,15,16,24,25,26,27], while also influencing skeletal remodeling through altered microvascular supply and inflammatory signaling [17,28,32,33,34,35,36]. In this context, the endothelium functions not merely as a target of injury but as a dynamic mediator of inter–organ communication.

The autonomic nervous system further integrates these axes through neuroimmune and neurovascular mechanisms. Sympathetic overactivation and vagal withdrawal influence intestinal permeability, vascular tone, and inflammatory responses [49,50,51,57], thereby linking the neurocardiac axis to both the heart–gut and heart–vascular pathways. Emerging evidence also suggests that neural signaling may modulate bone metabolism and osteokine release, extending autonomic influence to the heart–bone axis [36,49,57].

Finally, mitochondrial dysfunction and metabolic remodeling may represent a shared intracellular mechanism underlying multiorgan crosstalk. Impaired bioenergetics, oxidative stress, and altered substrate utilization have been observed across cardiac, endothelial, skeletal, and intestinal tissues in HF [20,21,22,23,24,61,62]. These changes may amplify systemic signaling loops, reinforcing the concept of HF as a network disease rather than a collection of isolated organ dysfunctions.

Collectively, these overlapping pathways highlight that the four axes described above converge on a limited number of shared biological processes, including inflammation, neurohormonal activation, endothelial dysfunction, and metabolic dysregulation [9,10,11,12,13,14,15,16,17,18,24,25,26,27,28,29,30,35,36,39,40,41,49,57,58]. Recognizing these common mechanisms provides a framework for identifying system-level therapeutic targets that may simultaneously modulate multiple organ interactions.

## 5. Therapeutic Implications: Targeting Multiorgan Crosstalk

Recognizing canine HF as a systemic disorder has important therapeutic implications. Traditional management strategies have primarily focused on hemodynamic optimization through diuretics, RAAS inhibition, and positive inotropic support. While these approaches remain foundational, they may not fully address the complex network of inter-organ interactions that contribute to disease progression [17,18,28,29,30].

The heart–gut axis represents a potential therapeutic target. In human HF, modulation of intestinal permeability, microbiota composition, and systemic inflammation has been proposed as an adjunct strategy to attenuate disease progression. Although direct interventional data in canine HF remain limited, the presence of gastrointestinal alterations and biomarker evidence of intestinal involvement suggest that targeting barrier dysfunction or inflammatory signaling pathways may influence systemic remodeling. Experimental in vivo models provide a controlled platform to evaluate whether pharmacologic modulation of gastrointestinal signaling, including histamine-mediated pathways, can alter cardiac outcomes [9,10,11,12,13,14,15,16,24,25,26,27].

Similarly, the heart–bone axis introduces endocrine and inflammatory mediators as potential contributors to cardiovascular remodeling. Circulating molecules such as OPG have been associated with vascular dysfunction and adverse cardiovascular outcomes in humans. Although the therapeutic modulation of bone-derived signaling in veterinary cardiology remains exploratory, understanding these pathways may inform future biomarker-guided or multi-targeted strategies [17,32,33,34,35,36].

The heart–vascular endothelium axis further underscores the need to consider vascular protection in HF management. Endothelial dysfunction contributes to impaired perfusion, inflammatory amplification, and organ vulnerability. Neurohormonal blockade partially mitigates these effects, yet persistent endothelial activation may sustain systemic injury. Experimental circulatory models have demonstrated that interventions targeting inflammatory and vasoactive pathways can influence endothelial responses, suggesting translational opportunities for refining therapeutic approaches [17,18,28,29,30].

Beyond pharmacologic modulation, non-pharmacologic strategies merit consideration within a multiorgan framework. Exercise-based interventions and neuromuscular stimulation have demonstrated beneficial effects on skeletal muscle metabolism, inflammatory tone, and functional capacity in human HF. Experimental systems allow controlled evaluation of whether such interventions influence systemic signaling pathways relevant to canine HF. Integrating these modalities into a systems-based perspective may facilitate individualized therapeutic strategies that extend beyond myocardial support alone [37,38,39,40,41,42].

## 6. Regenerative and Organelle-Based Therapeutic Perspectives

Recent advances in regenerative medicine introduce a system-level dimension to therapeutic strategies in HF. Mesenchymal stem cells (MSCs), including adipose-derived stem cells, exert immunomodulatory antifibrotic, and proangiogenic effects primarily through paracrine signaling rather than direct cardiomyocyte replacement. Experimental studies in both small- and large-animal cardiomyopathy models have demonstrated improvements in ventricular remodeling, attenuation of fibrosis, and modulation of inflammatory mediators following MSC administration. Importantly, these effects extend beyond myocardial tissue and may influence endothelial function, skeletal muscle metabolism, and systemic inflammatory tone—hallmarks of multiorgan cross-talk in HF. Although clinical evidence in veterinary cardiology remain limited and heterogeneous, preliminary reports suggest that adipose-derived stem cell therapy may improve functional parameters and quality-of-life indices in dogs in cardiomyopathy. Within a multiorgan framework, MSC therapy should therefore be evaluated not solely for myocardial recovery but for its capacity to modulate gut-derived inflammation, endothelial activation, and neurohormonal signaling. Beyond cell-based therapies, mitochondrial transplantation has emerged as an innovative experimental strategy to restore bioenergetic function in injured myocardium. Given that mitochondrial dysfunction represents a shared pathological mechanism across cardiac, endothelial, and skeletal tissues in HF, organelle-based therapies may exert systemic metabolic effects. Preclinical studies have demonstrated that exogenous mitochondrial transfer can improve ATP production, reduce oxidative stress, and enhance contractile recovery following ischemia injury. Within the context of cardiomyopathy models, mitochondrial transplantation offers a mechanistic platform to determine whether restoring cellular bioenergetics can attenuate multiorgan remodeling and biomarker shifts, including indices of intestinal injury (I–FABP), endothelial activation, and bone-derived signaling mediators [9,10,11,12,13,14,15,16,17,18,24,25,26,27,28,29,30,32,33,34,35,36,37,38,39,40,41,43,44,45,46,47,48,59,60,61,62,66,67,68].

In veterinary medicine, regenerative and organelle-based therapies remain exploratory and are not yet integrated into standard HF management. However, conceptualizing HF as a multiorgan network disorder suggests that regenerative interventions may function as systemic modulators rather than localized myocardial repair strategies. Future experimental studies should therefore incorporate multiorgan endpoints—including autonomic function, endothelial biomarkers, and gut permeability indices—when evaluating regenerative therapies. This perspective aligns with the emerging paradigm of precision and regenerative veterinary cardiology [9,10,11,12,13,14,15,16,17,18,19,24,25,26,27,28,29,30,42,43,44,45,46,47,48,50,51,52,53,54,55,56,57,58].

## 7. Future Directions and Translational Perspectives

Reframing canine HF as a multiorgan network disorder highlights the need for a shift toward integrative phenotyping. Future studies may benefit from longitudinal designs that incorporate cardiac imaging, circulating biomarkers, indices of intestinal barrier integrity (e.g., I–FABP), endothelial activation markers, endocrine mediators such as OPG, and autonomic function assessed by HRV. Such multimodal approaches may help clarify temporal relationships among organ systems and determine whether extracardiac alterations represent early drivers or secondary consequences of myocardial remodeling [9,10,11,12,13,14,15,16,17,18,19,24,25,26,27,28,29,30,32,33,34,35,36,37,38,39,40,41,42,50,51,52,53,54,55,56,57,58].

Naturally occurring canine MMVD and DCM provide valuable platforms for prospective investigation, particularly in preclinical and early-stage disease. Integration of clinical observations with controlled in vivo models, including anthracycline-induced cardiomyopathy and surgical remodeling systems, may facilitate more precise evaluation of multiorgan signaling pathways [4,5,6,7,8,19,20,21,22,23,31,37,38,39,40,41,49]. The development of composite biomarker panels incorporating indices of gut permeability, endothelial stress, autonomic imbalance, and skeletal signaling may improve risk stratification and disease monitoring. However, further validation across diverse populations will be required [9,10,11,12,13,14,15,16,17,18,19,24,25,26,27,28,29,30,50,51,52,53,54,55,56,57,58].

In parallel, advances in regenerative and organelle-based therapies, including mesenchymal stem cell approaches and mitochondrial-targeted strategies, warrant continued investigation. Future studies incorporating multiorgan endpoints may help determine whether these interventions modulate systemic pathways in addition to myocardial function [17,18,19,28,29,30,43,44,45,46,47,48,50,51,52,53,54,55,56,57,58,59,60,61,62,66,67,68]. Overall, continued integration of clinical and experimental approaches may support a more comprehensive understanding of HF as a multiorgan condition and contribute to the refinement of translational strategies in veterinary cardiology.

## 8. Conclusions

HF should no longer be viewed solely as a disorder of impaired myocardial contractility. Rather, accumulating clinical and experimental evidence supports its conceptualization as a dynamic multiorgan network disorder involving bidirectional interactions among the heart, intestine, vascular endothelium, skeletal system, and autonomic nervous system. These interconnected axes are unified by shared mechanisms including neurohormonal activation, oxidative stress, inflammatory signaling, and metabolic dysregulation. Naturally occurring canine HF, particularly MMVD and DCM, provides a uniquely valuable translational platform in which systemic remodeling unfolds within an intact physiological environment. When integrated with mechanistic in vivo models, this framework enables temporal dissection of organ-to-organ signaling and identification of network-level therapeutic targets [4,5,6,7,8,17,18,19,20,21,22,23,28,29,30,31,42,49,50,51,52,53,54,55,56,57,58,59,60,61,62]. Reframing canine HF as a multiorgan network disease represents more than a semantic shift.

It challenges traditional organ-centered paradigms and emphasizes the interconnected regulatory architecture underlying disease progression [42]. By integrating spontaneous canine heart disease with mechanistic in vivo models and emerging regenerative strategies, veterinary cardiology is uniquely positioned to contribute to comparative cardiovascular science. This system-based paradigm may ultimately redefine not only therapeutic strategy but also the conceptual boundaries between veterinary and human cardiovascular medicine [36,37,38,40,41,43,44,45,47,48,60,66].

## Data Availability

No new data were created or analyzed in this study. Data sharing is not applicable to this article.

## Abbreviations

The following abbreviations are used in this manuscript:

ACVIM	American College of Veterinary Internal Medicine
CPB	Cardiopulmonary bypass
DCM	Dilated cardiomyopathy
ET–1	Endothelin–1
HF	Heart failure
HRV	Heart rate variability
I–FABP	Intestinal-fatty acid binding protein
MMVD	Myxomatous mitral valve disease
MSC	Mesenchymal stem cell
OPG	Osteoprotegerin
OSTN	Osteocrin
RAAS	Renin–angiotensin–aldosterone system
